# Association between estimated glucose disposal rate and subclinical cerebrovascular atherosclerotic burden in non-diabetic middle-aged and older adults

**DOI:** 10.3389/fneur.2026.1946802

**Published:** 2026-10-20

**Authors:** Lei Guo, Yubo Zhao, Meng Qu, Ming Li, Jianchun Zhu, Sha Zheng, Jun Zhang

**Affiliations:** 1 Department of Neurology, Xindu District People’s Hospital of Chengdu, Chengdu, China; 2 Shi Ban Tan Community Healthcare Center, Chengdu, China; 3 Department of Endocrinology, Xindu District People’s Hospital of Chengdu, Chengdu, China

**Keywords:** estimated glucose disposal rate, insulin resistance, middle-aged and older adults, non-diabetic, subclinical cerebrovascular atherosclerotic burden

## Abstract

**Objective:**

To evaluate the association and dose–response relationship between estimated glucose disposal rate (eGDR), a surrogate marker of insulin resistance, and subclinical cerebrovascular atherosclerotic burden in non-diabetic middle-aged and older adults.

**Methods:**

This retrospective analysis used data from a prospective registry and included 604 adults aged ≥45 years without diabetes. Participants with symptomatic cerebrovascular disease, including prior ischemic stroke or transient ischemic attack attributable to cerebrovascular stenosis, were excluded. Cerebrovascular atherosclerosis was assessed using carotid duplex ultrasonography, head and neck magnetic resonance angiography, or head and neck computed tomography angiography. Based on neuroimaging results, participants were classified into three categories: no atherosclerosis, single vascular bed involvement, and dual vascular bed involvement. Associations between eGDR and atherosclerotic burden were evaluated using ordinal logistic regression models, including unadjusted, age- and sex-adjusted, and fully adjusted models. Restricted cubic spline (RCS) analysis was performed to examine the dose–response relationship.

**Results:**

All 604 participants were community-dwelling adults without symptomatic cerebrovascular disease. The mean age was 64 ± 11 years, and 220 (36.4%) were male. Participants with a higher subclinical cerebrovascular atherosclerotic burden were older, had a higher prevalence of hypertension, and had lower eGDR levels (*p* < 0.001). The prevalence of single and dual vascular bed involvement decreased progressively across eGDR quartiles, from the lowest (Q1) to the highest (Q4) quartile (single bed: 33.1% vs. 19.2%; dual beds: 13.2% vs. 2.0%). In the fully adjusted ordinal logistic regression model, each 1-SD increase in eGDR was associated with lower odds of being in a higher atherosclerotic burden category (OR = 0.74, 95% CI: 0.62–0.89, *p* = 0.001). Compared with Q1, participants in Q4 had lower odds of being in a higher burden category (OR = 0.42, 95% CI: 0.24–0.72, *p* = 0.001; P for trend = 0.002). The RCS analysis showed a linear correlation between eGDR and atherosclerotic burden (overall *p* = 0.002, non-linear *p* = 0.322).

**Conclusion:**

Among middle-aged and older adults without diabetes, lower eGDR was independently associated with a higher subclinical cerebrovascular atherosclerotic burden, with a linear pattern. These findings warrant further investigation in prospective studies.

## Introduction

Stroke continues to be a major cause of mortality and prolonged disability globally, with ischemic stroke making up over 80% of all instances (1). Cerebrovascular atherosclerosis is a key pathological substrate of ischemic stroke, and both its extent and severity are closely associated with the risks of stroke occurrence, recurrence, cognitive decline, and poor long-term outcomes, thereby imposing a substantial health and socioeconomic burden (2). Increasing evidence indicates that atherosclerosis is a systemic vascular disease that frequently involves multiple arterial beds simultaneously. Compared with isolated vascular lesions, cerebrovascular atherosclerotic burden, which reflects the extent of involvement across multiple vascular territories, provides a more comprehensive measure of atherosclerotic involvement and has been associated with an increased risk of recurrent ischemic events, adverse vascular outcomes, and poor prognosis (3–5). Therefore, understanding factors associated with cerebrovascular atherosclerotic burden may provide further insight into the vascular manifestations of atherosclerosis.

Insulin resistance (IR), characterized by impaired insulin-mediated glucose uptake and utilization, is a fundamental pathophysiological feature of metabolic dysfunction. Accumulating evidence indicates that IR promotes the initiation and progression of atherosclerosis via various mechanisms, such as endothelial dysfunction, persistent inflammation, oxidative stress, and the proliferation of vascular smooth muscle cells (6, 7). Notably, IR-related vascular injury often precedes the clinical onset of diabetes by years or even decades. With advancing age, insulin sensitivity progressively declines, and many middle-aged and older adults without diabetes already exhibit metabolic abnormalities and subclinical vascular damage despite not meeting the diagnostic criteria for diabetes (8, 9). However, whether IR-related metabolic status is associated with a higher burden of multi-bed cerebrovascular atherosclerosis in the non-diabetic population, and whether such an association follows a dose–response pattern, remains largely unknown.

Despite being the gold standard for measuring IR, the hyperinsulinemic-euglycemic clamp is too invasive and labor-intensive for widespread clinical screenings (10). Importantly, in the non-diabetic phase, regularly checked metabolic markers like fasting plasma glucose and glycated hemoglobin often appear normal, leading to a major diagnostic oversight where individuals at high risk with severe vascular damage go unnoticed in clinical settings (11). The estimated glucose disposal rate (eGDR) emerged as a validated, non-invasive alternative marker that uniquely integrates a history of hypertension, obesity metrics, and glycated hemoglobin. By merging hemodynamic load, visceral adiposity, and subtle glycemic fluctuations, eGDR sensitively captures latent IR (12). While a lower eGDR is linked to adverse cardiovascular events and poor stroke outcomes (13, 14), direct clinical evidence linking it to the comprehensive subclinical cerebrovascular atherosclerotic burden—particularly multi-bed involvement—remains scarce, especially within the critical early-intervention window of non-diabetic middle-aged and older populations.

Accordingly, using data from a prospective registry, we conducted a retrospective analysis of middle-aged and older adults without diabetes to investigate the association between eGDR and subclinical cerebrovascular atherosclerotic burden, as well as its dose–response relationship.

## Method

### Study design and population

The study was a cross-sectional analysis incorporated into the E-CHAMP (Evaluation of eGDR in Cardiovascular and Cerebrovascular Health Assessment and Management Program), a prospective registry program initiated in 2024 by the Sichuan Preventive Medicine Association. Relying on advanced medical equipment and telemedicine technology, the E-CHAMP project was designed to construct a standardized and reproducible stroke prevention and management platform.

Between 2024 and 2026, the registry consecutively recruited community-dwelling residents from eight distinct communities in the Xindu District of Chengdu, Sichuan, China. The primary inclusion criteria for the registry were individuals aged 45 years or older without a prior or current clinical diagnosis of diabetes mellitus. Diabetes status was confirmed by medical history, medication records, and baseline laboratory assessment. Participants were eligible if they were aged 45 years or older, had no prior or current diagnosis of diabetes mellitus, and had complete clinical, laboratory, anthropometric, and cerebrovascular imaging data. Participants were excluded if they had symptomatic cerebrovascular disease, including prior ischemic stroke or transient ischemic attack attributable to cerebrovascular stenosis; a history of coronary artery disease; severe hepatic or renal dysfunction; psychiatric disorders; incomplete key clinical information; or missing vascular imaging data required for assessment of cerebrovascular atherosclerotic burden.

The Ethics Committee of Chengdu Xindu District People’s Hospital approved the study protocol (Approval No. [IIT]PJ2025017), and it was conducted in strict adherence to the ethical principles of the Declaration of Helsinki. The prospective registry was formally registered in the Chinese Clinical Trial Registry (ChiCTR2500112202). Written informed consent was obtained from all participants before data collection.

### Data collection and measurements

Baseline data, including demographic characteristics, anthropometric measurements, clinical history, laboratory assessments, and cerebrovascular imaging data, were collected by trained clinical research coordinators using standardized questionnaires and protocols.

Demographic and clinical data, such as age, sex, smoking status, alcohol consumption, and medical history of hypertension, hyperlipidemia, and hyperhomocysteinemia, were obtained through structured face-to-face interviews and verified using electronic medical records whenever available.

Measurements of anthropometric data were taken with participants in light clothing and without shoes. The waist circumference (WC) was measured at the midpoint between the lowest rib and the iliac crest, while the hip circumference (HC) was measured at the fullest part of the buttocks. The height and weight were measured to an accuracy of 0.1 cm and 0.1 kg, respectively, and the body mass index (BMI) was calculated by dividing the weight in kilograms by the square of the height in meters. Using an automated electronic sphygmomanometer, blood pressure was measured after participants had rested in a seated position for at least 15 min.

After fasting for at least 8 h overnight, venous blood samples were collected from all participants in the morning. Glycated hemoglobin (HbA1c), fasting plasma glucose (FPG), total cholesterol (TC), triglycerides (TG), high-density lipoprotein cholesterol (HDL-C), low-density lipoprotein cholesterol (LDL-C), alanine aminotransferase (ALT), aspartate aminotransferase (AST), uric acid (UA), serum creatinine (Cr), urea, white blood cell count (WBC), red blood cell count (RBC), hemoglobin (Hb), and platelet count (PLT) were measured in the central laboratory of Chengdu Xindu District People’s Hospital using standardized laboratory procedures.

The formula used to calculate eGDR, a validated surrogate measure of insulin resistance, is:

eGDR=24.31−12.22×WHR−3.29×hypertension−0.57×HbA1c

Where hypertension was coded as 1 for participants with hypertension and 0 otherwise. Higher eGDR scores reflect better insulin sensitivity, whereas lower scores denote more insulin resistance.

### Assessment of cerebrovascular atherosclerotic burden

The primary outcome of this study was subclinical cerebrovascular atherosclerotic burden. Cerebrovascular atherosclerosis was assessed using a standardized multimodal vascular imaging protocol, including carotid duplex ultrasonography, head and neck MRA, and head and neck CTA, as appropriate according to the study protocol and clinical practice. These complementary imaging modalities were used to assess both intracranial and extracranial arterial beds. Two experienced neurologists independently assessed all imaging studies while blinded to participants’ clinical and laboratory data. Any differences were reconciled by consensus.

The intracranial vascular bed included the intracranial internal carotid, middle cerebral, anterior cerebral, posterior cerebral, vertebral, and basilar arteries. The extracranial vascular bed included the common carotid, extracranial internal carotid, vertebral, and subclavian arteries. Vascular bed involvement was defined as the presence of atherosclerotic plaque and/or arterial stenosis within the corresponding vascular bed.

Cerebrovascular atherosclerosis was assessed based on how many vascular beds were impacted. One point was assigned for the presence of atherosclerotic lesions in each vascular bed, resulting in a vascular bed involvement score ranging from 0 to 2. Participants were categorized into three mutually exclusive groups: no atherosclerosis (score = 0), defined as the absence of atherosclerotic plaque or arterial stenosis in both vascular beds; single vascular bed involvement (score = 1), defined as atherosclerotic lesions confined to either the intracranial or extracranial vascular bed; and dual vascular bed involvement (score = 2), defined as concurrent atherosclerotic lesions involving both intracranial and extracranial vascular beds.

### Statistical analysis

Statistical analyses were executed with R software (version 4.4.2; R Foundation for Statistical Computing, Vienna, Austria), considering a two-sided *p* value below 0.05 as statistically significant.

The Shapiro–Wilk test was used to evaluate the normality of continuous variables. Continuous variables that followed a normal distribution are shown as mean ± standard deviation (SD) and were compared across the three groups with subclinical cerebrovascular atherosclerotic burden using one-way ANOVA. For non-normally distributed variables, data are presented as median (interquartile range [IQR]) and analyzed with the Kruskal–Wallis test. Categorical data are shown as number (percentage) and compared using the *χ*^2^ test or Fisher’s exact test. To assess potential differences related to missing data, baseline characteristics were compared between participants included in the analysis and those excluded because of missing anthropometric or imaging data. Continuous variables were compared using the Student’s t test or Wilcoxon rank-sum test, as appropriate, and categorical variables were compared using the *χ*^2^ test or Fisher’s exact test, as appropriate.

To visualize the distribution of subclinical cerebrovascular atherosclerotic burden across different eGDR levels, participants were categorized into quartiles according to baseline eGDR values, and the proportions of participants with no atherosclerosis, single vascular bed involvement, and dual vascular bed involvement were displayed using stacked bar charts.

Ordinal logistic regression models were used to assess the association between eGDR and the subclinical cerebrovascular atherosclerotic burden, with subclinical cerebrovascular atherosclerotic burden treated as an ordinal outcome variable comprising three categories (no atherosclerosis, single vascular bed involvement, and dual vascular bed involvement). The proportional odds assumption was assessed before model fitting and was considered satisfied. eGDR was analyzed both as a continuous variable (per 1-SD increment) and in quartiles, with the lowest quartile (Q1) serving as the reference category. Three models with increasing adjustments were developed: Model 1 was unadjusted; Model 2 was adjusted for age and gender; and Model 3 was further adjusted for smoking, alcohol consumption, ALT, AST, UA, TG, TC, HDL-C, and LDL-C. Covariates were prespecified based on previous literature and clinical relevance, particularly factors considered potential confounders in studies examining the associations of eGDR with cardiovascular and atherosclerotic outcomes (15, 16). Because hypertension is incorporated into the conventional eGDR equation, it was not additionally included as a covariate in the primary multivariable models to avoid adjusting for a component of the exposure itself. Linear trend tests across eGDR quartiles were conducted by treating the median value of each quartile as a continuous variable.

To evaluate the robustness of the primary findings, we performed several sensitivity analyses. First, we used binary logistic regression, classifying participants with either single or dual vascular bed involvement as having cerebrovascular atherosclerosis and comparing them with those without the condition, with the same sequential adjustment strategy applied. Second, to examine the individual contributions of the eGDR components, hypertension, WHR, and HbA1c were entered separately into models adjusted for the same covariates as in Model 3. Third, to investigate whether the observed interaction with hypertension status could be partially attributable to the incorporation of hypertension into the eGDR equation, we recalculated a modified eGDR excluding the hypertension component (24.31–12.22*WHR - 0.57* HbA1c). This modified eGDR was standardized to a 1-SD increment, and the interaction analysis was repeated using the same covariate adjustment as in the primary model.

Potential dose–response and non-linear connections between eGDR and subclinical cerebrovascular atherosclerotic burden were investigated using restricted cubic spline regression, grounded in a fully adjusted ordinal logistic regression model. At the 5th, 35th, 65th, and 95th percentiles of the eGDR distribution, four knots were placed. The median eGDR value was used as a reference, and adjusted odds ratios (ORs) with 95% confidence intervals (CIs) were estimated across the continuous range. Wald tests were used to assess both overall and non-linear associations.

Prespecified subgroup analyses were performed according to age (<65 vs. ≥65 years), sex, body mass index (<24 vs. ≥24 kg/m^2^), hypertension (yes vs. no), smoking status (yes vs. no), and LDL-C (<2.6 vs. ≥2.6 mmol/L). Potential effect modification was assessed by including interaction terms between eGDR and each prespecified subgroup variable in the fully adjusted ordinal logistic regression model. All subgroup analyses were adjusted for the same covariates as Model 3, except for the stratification variable itself.

## Results

### Study population and baseline characteristics

A total of 800 community-dwelling middle-aged and older adults without diabetes were enrolled in the E-CHAMP registry between 2024 and 2026. After excluding 196 participants with missing cerebrovascular imaging examinations and incomplete anthropometric components required for eGDR calculation, 604 participants were included. Among them, 383 (63.4%) had no evidence of cerebrovascular atherosclerosis, 164 (27.2%) had single vascular bed involvement, and 57 (9.4%) had dual vascular bed involvement (Figure 1). Baseline characteristics of participants included in and excluded from the analysis were compared in Supplementary Table S1. Overall, the two groups showed broadly comparable baseline characteristics.

**Figure 1:**
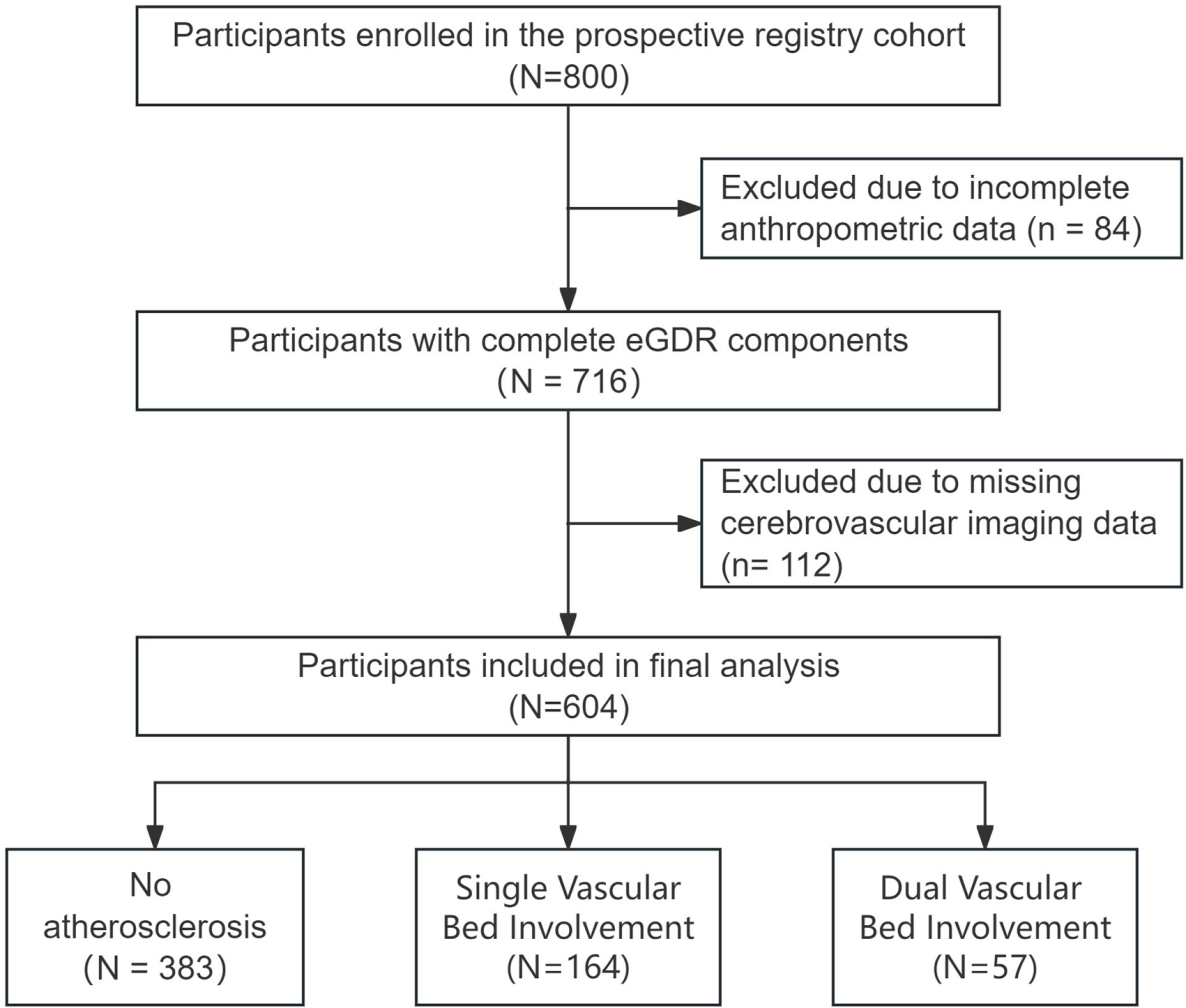
Study flow diagram of participant recruitment and selection.

The baseline characteristics, including demographic, clinical, and laboratory characteristics, of the 604 participants included in the analysis were summarized according to subclinical cerebrovascular atherosclerotic burden: no atherosclerosis (*n* = 383), single vascular bed involvement (*n* = 164), and dual vascular bed involvement (Table 1). Participants exhibiting a higher subclinical cerebrovascular atherosclerotic burden were older and presented with a greater prevalence of hypertension (*p* < 0.001). Baseline levels of eGDR decreased significantly as the extent of cerebrovascular atherosclerotic involvement increased, with the lowest eGDR levels observed in the dual vascular bed involvement group (*p* < 0.001).

**Table 1:** Baseline characteristics according to subclinical cerebrovascular atherosclerotic burden.

Characteristic	No atherosclerosis (*N* = 383)	Single vascular bed (*N* = 164)	Dual vascular beds (*N* = 57)	*p*-value
Demographics and anthropometrics				
Age (years)	63 ± 11	67 ± 11	69 ± 10	<0.001
Male (%)	135 (35%)	69 (39%)	16 (43%)	0.38
Height (cm)	157 ± 10	157 ± 13	157 ± 10	0.86
Weight (kg)	63 ± 34	62 ± 12	61 ± 11	0.76
BMI (kg/m^2^)	24.0 (22.1–26.3)	24.8 (22.5–26.7)	24.7 (23.0–26.4)	0.12
WC (cm)	86 (81–92)	87 (81–94)	87 (81–94)	0.19
HC (cm)	95 (90–100)	96 (89–101)	96 (91–99)	0.88
Clinical history *n* (%)				
Smoking	99 (25%)	52 (30%)	9 (24%)	0.55
Alcohol consumption	82 (21%)	39 (22%)	8 (22%)	0.95
Hypertension	174 (45%)	113 (64%)	23 (62%)	<0.001
Hyperlipidemia	176 (45%)	74 (42%)	12 (32%)	0.31
Hyperhomocysteine	98 (25%)	40 (23%)	5 (14%)	0.27
Key metabolic index				
eGDR	9.11 (6.60–10.08)	6.95 (6.17–9.46)	7.46 (6.44–9.21)	<0.001
Laboratory measurements				
HbA1c (%)	5.80 (5.50–6.00)	5.75 (5.60–6.00)	5.70 (5.50–6.00)	0.70
FPG (mmol/L)	5.60 (5.00–6.70)	5.80 (5.20–6.90)	5.60 (4.80–6.40)	0.16
TG (mmol/L)	1.55 (1.06–2.32)	1.53 (1.11–2.41)	1.38 (0.82–2.02)	0.27
TC (mmol/L)	5.05 ± 1.10	4.85 ± 1.04	4.53 ± 1.03	0.01
HDL (mmol/L)	1.46 (1.24–1.71)	1.45 (1.22–1.66)	1.40 (1.20–1.61)	0.45
LDL (mmol/L)	3.01 ± 0.78	2.88 ± 0.78	2.65 ± 0.70	0.01
WBC (×10*9/L)	5.84 (4.66–7.23)	6.14 (4.89–7.78)	6.11 (5.32–6.89)	0.10
RBC (×10*12/L)	4.39 (4.03–4.76)	4.39 (4.02–4.72)	4.38 (3.80–4.63)	0.48
Hb (g/L)	133 (122–143)	131 (119–142)	131 (118–148)	0.65
PLT (×10*9/L)	175 (137–212)	164 (135–204)	162 (113–201)	0.21
ALT (U/L)	21 (16–30)	20 (16–29)	21 (15–27)	0.44
AST (U/L)	25 (21–31)	24 (21–30)	29 (22–32)	0.28
UA (μmol/L)	295 (251–351)	303 (245–363)	312 (246–382)	0.49
Cr (μmol/L)	64 (56–78)	68 (58–80)	68 (61–80)	0.06

Data are presented as mean ± SD, median (IQR), or number (%). *p*-values were calculated using one-way analysis of variance or the Kruskal–Wallis test for continuous variables and the *χ*^2^ test or Fisher’s exact test for categorical variables, as appropriate.

BMI, body mass index; WC, waist circumference; HC, hip circumference; eGDR, estimated glucose disposal rate; HbA1c, glycated hemoglobin A1c; FPG, fasting plasma glucose; TG, triglycerides; TC, total cholesterol; HDL, high-density lipoprotein cholesterol; LDL, low-density lipoprotein cholesterol; WBC, white blood cell count; RBC, red blood cell count; Hb, hemoglobin; PLT, platelet count; ALT, alanine aminotransferase; AST, aspartate aminotransferase; UA, uric acid; Cr, serum creatinine.

### Distribution of subclinical cerebrovascular atherosclerotic burden across eGDR quartiles

To visualize the distribution of subclinical cerebrovascular atherosclerotic burden across different levels of eGDR, participants were categorized into quartiles based on baseline eGDR values. As illustrated by the stacked bar charts (Figure 2), a lower prevalence of both single and dual vascular bed involvement was observed in the higher eGDR quartiles. Specifically, the prevalence of single vascular bed involvement declined from 33.1% in Q1 to 19.2% in Q4, while the prevalence of dual vascular bed involvement dropped sharply from 13.2% in Q1 to merely 2.0% in Q4.

**Figure 2:**
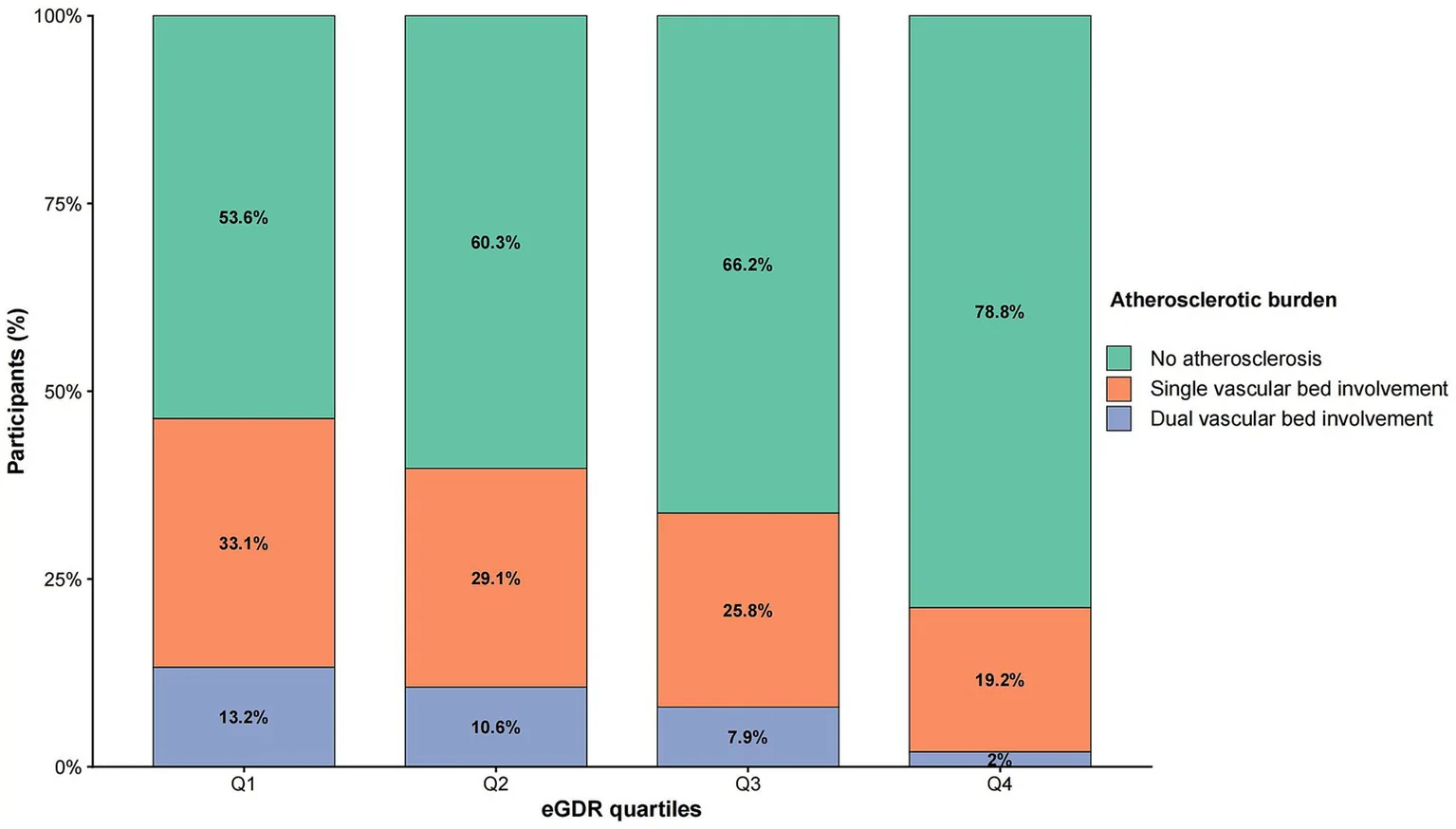
Distribution of subclinical cerebrovascular atherosclerotic burden across eGDR quartiles. The stacked bar chart illustrates the percentage of participants with different stages of subclinical cerebrovascular atherosclerotic burden (no disease, single vascular bed, and dual vascular beds) across the quartiles of eGDR. As eGDR levels increases from the lowest quartile (Q1) to the highest quartile (Q4), a significant graded decrease in the prevalence of both single and dual vascular bed atherosclerosis is observed, accompanied by a corresponding increase in the proportion of participants without disease. eGDR, estimated glucose disposal rate.

### Association between eGDR and subclinical cerebrovascular atherosclerotic burden

The associations evaluated via ordinal logistic regression models are presented in Table 2. In the unadjusted model (Model 1), higher eGDR was strongly associated with lower odds of being in a higher category of subclinical cerebrovascular atherosclerotic burden. This inverse association remained stable after adjusting for age and sex in Model 2. In the fully adjusted model (Model 3), which controlled for age, sex, smoking status, alcohol consumption, ALT, AST, UA, TC, TG, HDL-C, and LDL-C, each 1-SD increase in eGDR was independently associated with lower odds of being in a higher category of subclinical cerebrovascular atherosclerotic burden (OR = 0.74, 95% CI: 0.62–0.89, *p* = 0.001). When analyzed as a categorical variable, compared with participants in the lowest quartile (Q1), those in the highest eGDR quartile (Q4) had lower odds of being in a higher burden category (OR = 0.42, 95% CI: 0.24–0.72, *p* = 0.001; P for trend = 0.002).

**Table 2:** Association between eGDR and subclinical cerebrovascular atherosclerotic burden.

Model	OR per 1-SD increase in eGDR	95% CI	*p*-value
A. Association between eGDR and subclinical cerebrovascular atherosclerotic burden			
Model 1	0.67	0.56–0.79	<0.001
Model 2	0.73	0.61–0.87	<0.001
Model 3	0.74	0.62–0.89	0.001

eGDR quartiles	OR per 1-SD increase in eGDR	95% CI	*p*-value
B. Association between eGDR quartiles and subclinical cerebrovascular atherosclerotic burden			
Q1 (lowest)	Reference	—	—
Q2	0.87	0.55–1.38	0.56
Q3	0.69	0.43–1.11	0.13
Q4 (highest)	0.42	0.24–0.72	0.001
P for trend	—	—	0.002

Model 1: unadjusted. Model 2: adjusted for age and sex. Model 3: additionally adjusted for smoking, alcohol consumption, ALT, AST, UA, TC, TG, HDL, LDL. Odds ratios in panels A and B were estimated using ordinal logistic regression models, whereas odds ratios in panel C were estimated using binary logistic regression models. P for trend was calculated by modeling the median value of each eGDR quartile as a continuous variable.

### Dose–response relationship between eGDR and subclinical cerebrovascular atherosclerotic burden

In RCS regression analysis, eGDR exhibited a significant overall association with subclinical cerebrovascular atherosclerotic burden (P for overall = 0.002). The test revealed no deviation from linearity (P for non-linearity = 0.322), with the odds of being in a higher burden category decreasing progressively across the increasing spectrum of eGDR (Figure 3).

**Figure 3:**
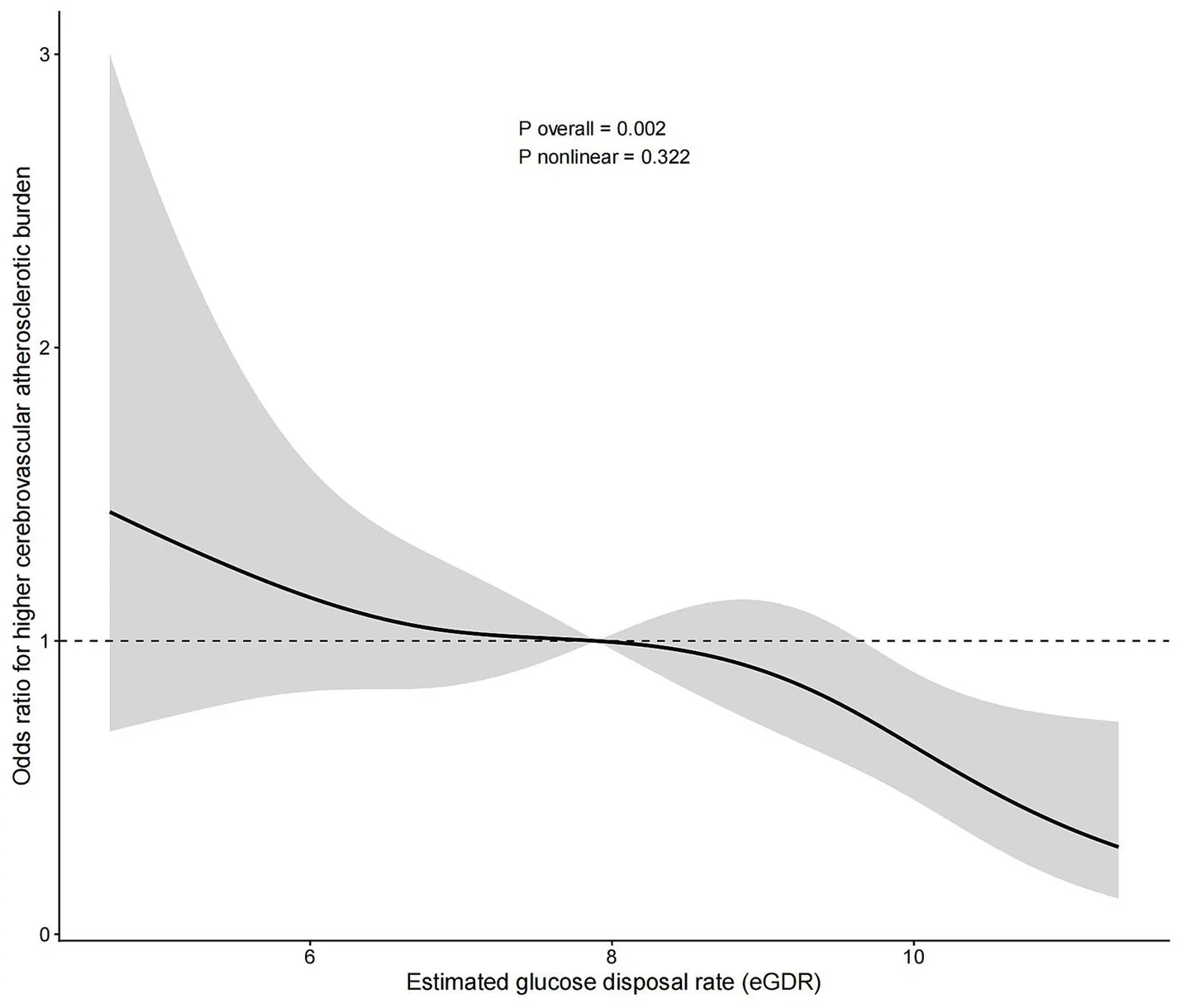
Restricted cubic spline curve for the association between eGDR and subclinical cerebrovascular atherosclerotic burden. The solid black line represents the estimated ORs for a higher subclinical cerebrovascular atherosclerotic burden category across the continuous spectrum of eGDR, adjusted for the covariates in Model 3. The grey shaded area indicates the 95% CIs. The reference point (OR = 1) is anchored at the median value of eGDR. A significant linear relationship was observed (P for overall = 0.002), with no evidence of non-linearity (P for non-linear = 0.322), demonstrating a steady decline in the risk of advanced atherosclerotic burden with increasing eGDR levels.

### Subgroup analyses and sensitivity analyses

Stratified analyses were conducted to evaluate the consistency of the link between a 1-SD rise in eGDR and subclinical cerebrovascular atherosclerotic burden (Figure 4). The inverse association between eGDR and subclinical cerebrovascular atherosclerotic burden remained generally consistent across most subgroups. A significant interaction was observed for hypertension status (P for interaction = 0.003), whereas no significant interactions were detected for age, sex, BMI, smoking status, or LDL-C level (all P for interaction >0.05).

**Figure 4:**
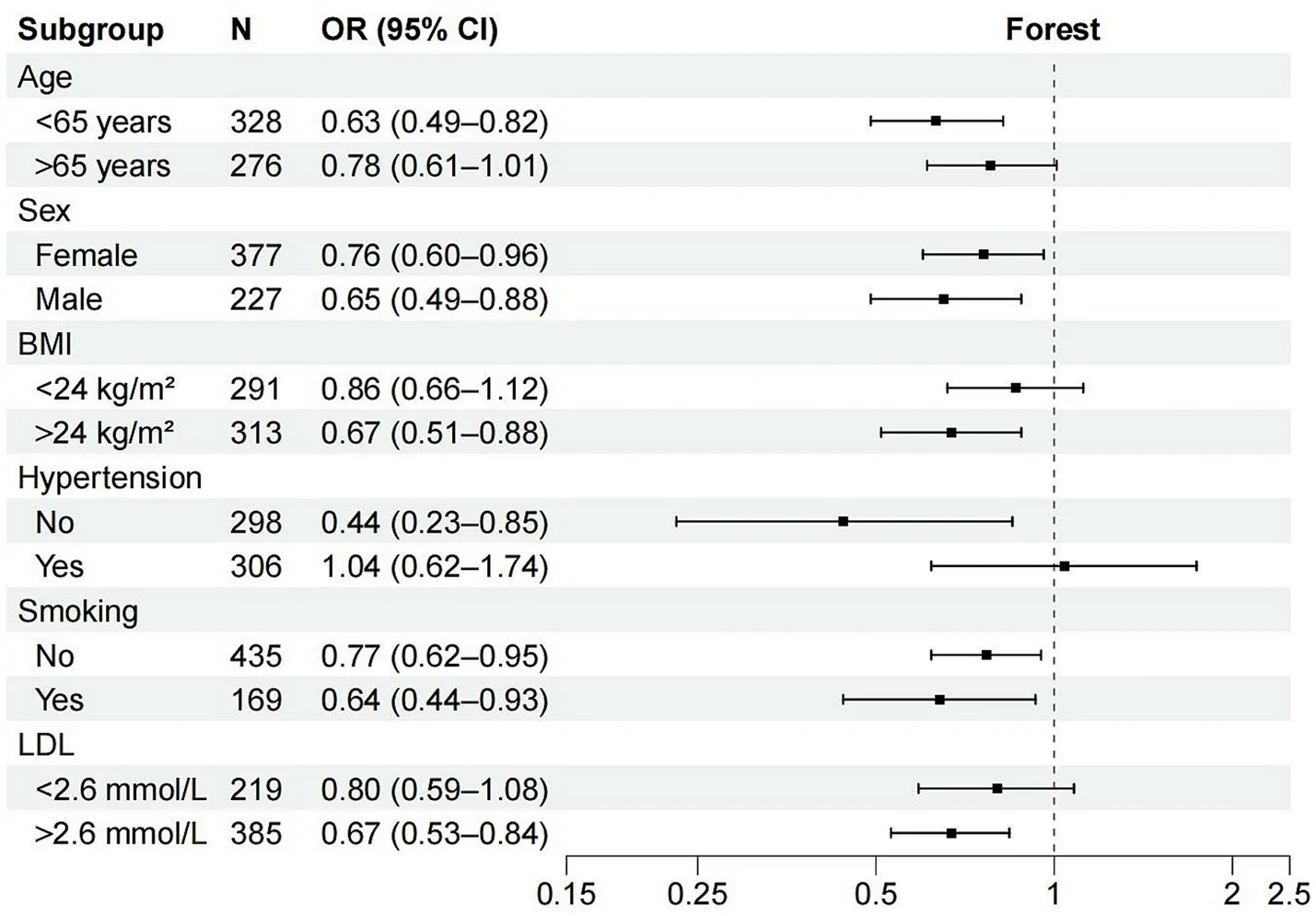
Subgroup analysis of the association between eGDR (per 1-SD increase) and subclinical cerebrovascular atherosclerotic burden. The forest plot displays the adjusted ORs and 95% CIs for a higher subclinical cerebrovascular atherosclerotic burden category per 1-SD increase in eGDR, stratified by age, sex, BMI, hypertension, smoking status, and LDL levels. All subgroup models were adjusted for the covariates in Model 3 (except the stratified variable itself). BMI, body mass index; CI, confidence interval; eGDR, estimated glucose disposal rate; LDL, low-density lipoprotein cholesterol; OR, odds ratio; SD, standard deviation.

In stratified analyses, higher eGDR was associated with lower subclinical cerebrovascular atherosclerotic burden among participants without hypertension (OR = 0.44, 95% CI: 0.23–0.85, *p* = 0.014), whereas no association was observed among those with hypertension (OR = 1.04, 95% CI: 0.62–1.74, *p* = 0.892). The inverse association remained significant among participants with BMI ≥ 24 kg/m^2^ (OR = 0.67, 95% CI: 0.51–0.88, *p* = 0.004) and LDL-C ≥ 2.6 mmol/L (OR = 0.67, 95% CI: 0.53–0.84, *p* < 0.001). For the other subgroup variables, including age, sex, and smoking status, the associations showed similar directions across strata.

Several sensitivity analyses were performed to assess the robustness of the findings. The inverse association between eGDR and cerebrovascular atherosclerosis remained significant when a binary outcome indicating the presence of any cerebrovascular atherosclerosis was used (fully adjusted OR = 0.72, 95% CI: 0.60–0.87, *p* < 0.001; Supplementary Table S2). When individual eGDR components were examined separately, hypertension was associated with a higher subclinical cerebrovascular atherosclerotic burden in both the hypertension-only model (OR = 1.80, 95% CI: 1.26–2.58, *p* = 0.001) and the component-wise model including hypertension, WHR, and HbA1c (OR = 1.79, 95% CI: 1.25–2.56, *p* = 0.001), whereas WHR and HbA1c were not significantly associated with burden (Supplementary Table S3). In addition, when eGDR was recalculated without the hypertension component, the interaction between modified eGDR and hypertension remained statistically significant (P for interaction = 0.002), with an inverse association observed among participants without hypertension (OR = 0.67, 95% CI: 0.50–0.89) but not among those with hypertension (OR = 1.17, 95% CI: 0.94–1.46; Supplementary Table S4).

## Discussion

This study systematically evaluated the association between the eGDR and subclinical cerebrovascular atherosclerotic burden in middle-aged and older adults without diabetes. We found that lower eGDR was independently associated with a higher subclinical cerebrovascular atherosclerotic burden, with a linear dose–response relationship observed across the eGDR distribution. Moreover, this association was observed among individuals without clinical diabetes, suggesting that metabolic alterations reflected by eGDR may be associated with subclinical cerebrovascular atherosclerotic burden before the development of overt diabetes. These findings support the relevance of insulin resistance-related metabolic status to subclinical cerebrovascular atherosclerosis in individuals without diabetes.

Previous studies have reported that lower eGDR is associated with cardiovascular events, ischemic stroke, and adverse clinical outcomes (13, 14). However, most of these investigations have focused on clinical vascular events rather than subclinical cerebrovascular atherosclerosis, and evidence in non-diabetic populations remains limited. Our findings extend previous observations by showing that lower eGDR was independently associated with a higher subclinical cerebrovascular atherosclerotic burden among middle-aged and older adults without diabetes. This finding suggests that insulin resistance-related metabolic dysfunction may be related to subclinical cerebrovascular atherosclerosis even in the absence of overt diabetes, providing further support for an association between insulin resistance-related metabolic status and cerebrovascular atherosclerosis.

Unlike most previous studies that focused on single vascular lesions, such as carotid plaques or intracranial arterial stenosis, our study used subclinical cerebrovascular atherosclerotic burden as the primary outcome, integrating both intracranial and extracranial vascular involvement to comprehensively characterize the extent of cerebrovascular atherosclerotic involvement across vascular territories. Growing evidence indicates that multi-bed vascular involvement reflects more advanced systemic atherosclerosis and is associated with a higher risk of recurrent stroke, cardiovascular events, and mortality (17, 18). Accordingly, a composite measure of cerebrovascular atherosclerotic burden may capture the distribution of atherosclerotic involvement across vascular territories more comprehensively than isolated vascular lesions (19). In this context, our finding that lower eGDR was independently associated with a greater subclinical cerebrovascular atherosclerotic burden suggests that insulin resistance-related metabolic status may be associated with a broader distribution of cerebrovascular atherosclerosis across vascular territories. This observation further expands current understanding of the relationship between insulin resistance and cerebrovascular disease.

Subgroup analyses further revealed that the inverse association between eGDR and subclinical cerebrovascular atherosclerotic burden was generally consistent across most prespecified clinical subgroups, with hypertension status exhibiting a significant effect modification. Among participants without hypertension, higher eGDR was associated with lower atherosclerotic burden, whereas no significant association was observed among those with hypertension.

Given that hypertension is an integral component of the traditional eGDR formula, the observed significant interaction by hypertension status warrants cautious interpretation. To assess whether this finding might be largely explained by the mathematical inclusion of hypertension in the eGDR formula, we repeated the analysis using a modified eGDR measure that excluded the hypertension component. The interaction between the modified eGDR and hypertension status remained statistically significant, suggesting that this modifying effect cannot be fully attributed to mathematical redundancy. Nevertheless, this sensitivity analysis does not establish a biological interaction or completely exclude the possibility of mathematical coupling related to the composite construction of eGDR. Therefore, the hypertension-specific interaction should be interpreted cautiously.

Importantly, the observed association between eGDR and subclinical cerebrovascular atherosclerotic burden should not be interpreted as independent of its individual components. In sensitivity analyses, hypertension was independently associated with a higher atherosclerotic burden, whereas WHR and HbA1c showed no significant associations after mutual adjustment. These findings suggest that the association captured by eGDR may reflect the contributions of its constituent metabolic components. Accordingly, eGDR may be viewed as a continuous composite surrogate of insulin resistance-related metabolic status that integrates information on hypertension, glycemic control, and central adiposity into a single measure. This composite structure allows several interrelated metabolic characteristics to be summarized by a single measure, but also limits the ability to disentangle the contribution of insulin resistance itself from that of its individual components. Whether eGDR adds incremental value beyond these components requires prospective validation.

The observed effect modification by hypertension may have plausible biological explanations. Insulin resistance-related metabolic abnormalities may influence vascular integrity through endothelial dysfunction, chronic inflammation, oxidative stress, and dysregulated lipid metabolism (20, 21). Meanwhile, hypertension-related hemodynamic load, abnormal shear stress, vascular remodeling, and structural changes in the arterial wall may also contribute to cerebrovascular atherosclerosis (22). Thus, the association between insulin resistance-related metabolic status and cerebrovascular atherosclerosis may differ according to hypertension status. However, these mechanisms remain speculative and cannot be established from our cross-sectional data. Further longitudinal and mechanistic studies are warranted to clarify the interplay between hypertension and insulin resistance-related metabolic status.

From the standpoint of clinical and public health, our findings provide cross-sectional evidence that lower eGDR is associated with a broader distribution of subclinical cerebrovascular atherosclerotic involvement across vascular beds. As a composite measure derived from routinely available clinical parameters, eGDR summarizes several interrelated metabolic characteristics on a continuous scale. However, the temporal relationship and clinical implications of this association cannot be determined from the present study and warrant further investigation in prospective longitudinal studies.

Several limitations of this study should be acknowledged. First, the cross-sectional design precludes determination of the temporal relationship between eGDR and subclinical cerebrovascular atherosclerotic burden and does not allow causal inference. Second, the study population was primarily composed of middle-aged and older community-dwelling adults from Southwest China, which may limit the generalizability of the findings to populations with different demographic, geographic, and metabolic characteristics. Differences in body composition, metabolic profiles, vascular risk factors, and healthcare systems may contribute to variation in eGDR levels and cerebrovascular atherosclerotic burden and may influence the observed association between them. Therefore, the magnitude and clinical relevance of our findings may not be directly applicable to populations with different characteristics or healthcare contexts. External validation in geographically diverse and ethnically heterogeneous populations is warranted.

Third, approximately one-quarter of initially eligible participants were excluded because of missing anthropometric or imaging data. Although included and excluded participants had broadly comparable baseline characteristics (Supplementary Table S1), selection bias cannot be completely excluded. Fourth, insulin resistance was not directly assessed using the hyperinsulinemic-euglycemic clamp, owing to the practical constraints of a large-scale community-based study. Instead, eGDR was used as a surrogate derived from routinely available clinical parameters. Thus, the findings should be interpreted as an association between the composite metabolic status reflected by eGDR and subclinical cerebrovascular atherosclerotic burden, rather than an association based on directly measured insulin resistance. In addition, the use of different imaging modalities may have introduced variability in lesion detection and outcome classification. Although predefined criteria were applied and imaging studies were independently reviewed by two experienced neurologists, measurement error and modality-related misclassification cannot be completely ruled out. Finally, although multiple demographic and metabolic confounders were adjusted for, residual confounding from unmeasured lifestyle, environmental, or genetic factors cannot be completely excluded.

In conclusion, our study indicates that, among middle-aged and older adults without diabetes, lower eGDR was independently associated with a higher subclinical cerebrovascular atherosclerotic burden, with a linear pattern across the eGDR distribution. These findings suggest that insulin resistance-related metabolic status, as reflected by eGDR, is associated with subclinical cerebrovascular atherosclerosis even in the absence of overt diabetes. Given the composite nature of eGDR, these findings should be interpreted in the context of its constituent metabolic factors. Further prospective studies are warranted to clarify the temporal relationship and determine whether eGDR provides information beyond its individual components.

## Data Availability

The datasets presented in this article are not readily available because all raw data is available from the corresponding author on reasonable request. Requests to access the datasets should be directed to 501838695@qq.com.
