# Supplementary material for: Association between estimated glucose disposal rate and subclinical cerebrovascular atherosclerotic burden in non-diabetic middle-aged and older adults
Source: Front Neurol. 2026 Oct 20;17:1946802. doi: 10.3389/fneur.2026.1946802 (PMC13727493; doi:10.3389/fneur.2026.1946802)
Supplement: Supplementary file 1 [file Table_1.DOCX]

**Supplementary Materials**

**Table of Contents**

[Supplementary Table S1. Baseline characteristics of included and excluded participants	2](#_Toc769)

[Supplementary Table S2. Sensitivity analyses of the association between eGDR and the presence of cerebrovascular atherosclerosis	3](#_Toc4027)

[Supplementary Table S3. Sensitivity analyses examining the associations of individual eGDR components with subclinical cerebrovascular atherosclerotic burden	4](#_Toc6826)

[Supplementary Table S4. Sensitivity analysis using eGDR recalculated without hypertension	5](#_Toc15478)

**Supplementary Table S1. Baseline characteristics of included and excluded participants**

| **Variable** | **Included (n=604)** | **Excluded (n=196)** | **P value** |
| --- | --- | --- | --- |
| Age (years) | 64.22 ± 11.14 | 63.77 ± 11.38 | 0.63 |
| Male (%) | 220 (36.4%) | 75 (38.3%) | 0.64 |
| Smoking (%) | 160 (26.5%) | 57 (29.1%) | 0.48 |
| Alcohol consumption (%) | 129 (21.4%) | 35 (17.9%) | 0.29 |
| Hypertension (%) | 310 (51.3%) | 87 (44.4%) | 0.09 |
| Hyperlipidemia (%) | 262 (43.4%) | 77 (39.3%) | 0.31 |
| Hyperhomocysteinemia (%) | 143 (23.7%) | 49 (25.0%) | 0.71 |
| HbA1c (%) | 5.80 (5.50–6.00) | 5.70 (5.50–6.00) | 0.11 |
| FPG (mmol/L) | 5.70 (5.00–6.70) | 5.80 (5.00–6.70) | 0.82 |
| TG (mmol/L) | 1.53 (1.07–2.32) | 1.50 (1.00–2.14) | 0.43 |
| TC (mmol/L) | 4.88 (4.19–5.65) | 4.83 (4.12–5.63) | 0.56 |
| HDL (mmol/L) | 1.45 (1.22–1.69) | 1.42 (1.25–1.67) | 0.95 |
| LDL (mmol/L) | 2.92 (2.39–3.42) | 2.84 (2.37–3.40) | 0.44 |
| WBC (×10^9/L) | 5.89 (4.78–7.43) | 6.04 (4.90–7.99) | 0.19 |
| RBC (×10^12/L) | 4.39 (4.02–4.74) | 4.37 (4.07–4.72) | 0.90 |
| Hb (g/L) | 132.00 (120.00–143.00) | 133.50 (124.00–142.00) | 0.15 |
| PLT (×10^9/L) | 170.50 (134.00–209.00) | 177.50 (141.50–212.25) | 0.40 |
| ALT (U/L) | 21.00 (16.00–30.00) | 20.00 (15.00–28.50) | 0.14 |
| AST (U/L) | 25.00 (21.00–31.00) | 24.00 (21.00–29.00) | 0.05 |
| UA (μmol/L) | 299.00 (249.00–357.00) | 301.00 (245.50–352.25) | 0.88 |
| Cr (μmol/L) | 65.70 (56.40–78.60) | 65.30 (55.58–79.00) | 0.89 |

**Note:** Participants were classified as included if they met all eligibility criteria and had complete data required for the primary analyses. Excluded participants met the eligibility criteria but were not included in the final analysis because of missing anthropometric data required for eGDR calculation or imaging data. Data are presented as mean ± SD, median (IQR), or number (%). P values were calculated using the Student’s t test or Wilcoxon rank-sum test for continuous variables and the χ² test or Fisher’s exact test for categorical variables, as appropriate.

*Abbreviations: HbA1c, glycated hemoglobin A1c; FPG, fasting plasma glucose; TG, triglycerides; TC, total cholesterol; HDL, high-density lipoprotein cholesterol; LDL, low-density lipoprotein cholesterol; WBC, white blood cell count; RBC, red blood cell count; Hb, hemoglobin; PLT, platelet count; ALT, alanine aminotransferase; AST, aspartate aminotransferase; UA, uric acid; Cr, serum creatinine.*

**Supplementary Table S2. Sensitivity analyses of the association between eGDR and the presence of cerebrovascular atherosclerosis**

| **Model** | **OR per 1-SD increase in eGDR** | **95% CI** | **P value** |
| --- | --- | --- | --- |
| Model 1 | 0.65 | 0.55–0.78 | <0.001 |
| Model 2 | 0.71 | 0.59–0.85 | <0.001 |
| Model 3 | 0.72 | 0.60–0.87 | <0.001 |

**Note:** In this sensitivity analysis, participants with either single or dual vascular bed involvement were classified as having cerebrovascular atherosclerosis, whereas those without vascular involvement were classified as having no cerebrovascular atherosclerosis. Associations were assessed using binary logistic regression. Model 1 was unadjusted; Model 2 was adjusted for age and sex; and Model 3 was further adjusted for smoking status, alcohol consumption, ALT, AST, UA, TC, TG, HDL-C, and LDL-C. eGDR was analyzed as a continuous exposure standardized to a 1-SD increment. ORs represent the odds of having any cerebrovascular atherosclerosis.

**Supplementary Table S3. Sensitivity analyses examining the associations of individual eGDR components with subclinical cerebrovascular atherosclerotic burden**

| **Model** | **Variable** | **OR (95% CI)** | **P** |
| --- | --- | --- | --- |
| eGDR model | eGDR per 1-SD | 0.74 (0.62–0.89) | 0.001 |
| Hypertension-only model | HBP | 1.80 (1.26–2.58) | 0.001 |
| Component-wise model | HBP | 1.79 (1.25–2.56) | 0.001 |
|  | WHR per 1-SD | 1.08 (0.90–1.28) | 0.412 |
|  | HbA1c per 1-SD | 0.93 (0.78–1.11) | 0.411 |

**Note:** The eGDR model included eGDR as a continuous exposure standardized to a 1-SD increment. The hypertension-only model included hypertension as a binary exposure, whereas the component-wise model included hypertension, WHR, and HbA1c as separate predictors, with WHR and HbA1c standardized to 1-SD increments. All models were adjusted for age, sex, smoking status, alcohol consumption, ALT, AST, UA, TC, TG, HDL-C, and LDL-C. Odds ratios were estimated using ordinal logistic regression and represent the odds of being in a higher category of subclinical cerebrovascular atherosclerotic burden.

**Supplementary Table S4. Sensitivity analysis using eGDR recalculated without hypertension**

| **Analysis** | **No hypertension OR (95% CI)** | **Hypertension OR (95% CI)** | **P for interaction** |
| --- | --- | --- | --- |
| Original eGDR | 0.44 (0.23–0.85) | 1.04 (0.62–1.74) | 0.003 |
| modified eGDR | 0.67 (0.50–0.89) | 1.17 (0.94–1.46) | 0.002 |

**Note:** ORs represent the odds of being in a higher category of subclinical cerebrovascular atherosclerotic burden per 1-SD increase in the corresponding eGDR measure. The original eGDR incorporates hypertension as a component, whereas the modified eGDR was recalculated without the hypertension component using the formula 24.31 − 12.22 × WHR − 0.57 × HbA1c. P values for interaction were obtained from the interaction terms between 1-SD standardized eGDR and hypertension status in the corresponding ordinal logistic regression models. All models were adjusted for age, sex, smoking status, alcohol consumption, ALT, AST, UA, TC, TG, HDL-C, and LDL-C.
